# Influence of Acute Complications on Outcome 3 Months after Ischemic Stroke

**DOI:** 10.1371/journal.pone.0075719

**Published:** 2013-09-24

**Authors:** Maike Miriam Grube, Hans-Christian Koennecke, Georg Walter, Andreas Meisel, Jan Sobesky, Christian Hans Nolte, Ian Wellwood, Peter Ulrich Heuschmann, on behalf of the Berlin Stroke Register (BSR)

**Affiliations:** 1 Center for Stroke Research Berlin (CSB), Klinik für Neurologie, Charité - Universitätsmedizin Berlin, Berlin, Germany; 2 Division of Health and Social Care Research, King’s College London, London, United Kingdom; 3 Vivantes Klinikum im Friedrichshain, Berlin, Germany; 4 Vivantes Klinikum Spandau, Berlin, Germany; 5 Institute of Clinical Epidemiology and Biometry, University of Würzburg, Center for Clinical Studies, University Hospital Würzburg, Comprehensive Heart Failure Center, University of Würzburg, Würzburg, Germany; University of Münster, Germany

## Abstract

**Background:**

Early medical complications are potentially modifiable factors influencing in-hospital outcome. We investigated the influence of acute complications on mortality and poor outcome 3 months after ischemic stroke.

**Methods:**

Data were obtained from patients admitted to one of 13 stroke units of the Berlin Stroke Registry (BSR) who participated in a 3-months-follow up between June 2010 and September 2012. We examined the influence of the cumulative number of early in-hospital complications on mortality and poor outcome (death, disability or institutionalization) 3 months after stroke using multivariable logistic regression analyses and calculated attributable fractions to determine the impact of early complications on mortality and poor outcome.

**Results:**

A total of 2349 ischemic stroke patients alive at discharge from acute care were included in the analysis. Older age, stroke severity, pre-stroke dependency and early complications were independent predictors of mortality 3 months after stroke. Poor outcome was independently associated with older age, stroke severity, pre-stroke dependency, previous stroke and early complications. More than 60% of deaths and poor outcomes were attributed to age, pre-stroke dependency and stroke severity and in-hospital complications contributed to 12.3% of deaths and 9.1% of poor outcomes 3 months after stroke.

**Conclusion:**

The majority of deaths and poor outcomes after stroke were attributed to non-modifiable factors. However, early in-hospital complications significantly affect outcome in patients who survived the acute phase after stroke, underlining the need to improve prevention and treatment of complications in hospital.

## Background

Despite achievements in early treatment, management and secondary prevention, a substantial proportion of stroke patients still experience severe consequences in terms of mortality and morbidity [1-8]. A recent comparison of variations in stroke consequences in different European regions reported that 40% of first-ever stroke patients suffer from poor outcome in terms of impairment, institutionalization or death 3 months after the index event [9]. Older age, stroke severity, comorbidity and pre-stroke dependency have consistently been reported as determinants for mortality and bad outcome after stroke [7,9-12]. Early complications like pneumonia and increased intracranial pressure have been shown to be independently associated with increased mortality and poor functional outcome [4,13-21]. We recently demonstrated that in-hospital complications account for up to 13-29% of in-hospital morbidity or mortality [4]. However, currently there is little evidence on the longer-term effects of early complications on stroke outcome. We aimed to examine the impact of early medical complications on mortality and poor outcome 3 months after ischemic stroke in patients discharged alive from acute care hospital.

## Methods

### The Berlin Stroke Register

The Berlin Stroke Register (BSR) is a network of stroke units in the city of Berlin (14 stroke units in June 2010) aiming to improve acute treatment of stroke patients [22]. The hospitals prospectively collect data for all patients with stroke or TIA in order to continuously monitor quality of stroke care. The participating stroke units currently document the treatment of about 9 000 patients a year, thereby covering an estimated 80% of stroke patients in Berlin. Data collected by members of the hospital staff include information on age, sex, stroke severity, functional impairment at stroke onset, diagnostic and therapeutic measures, early rehabilitation and secondary prevention, and early complications. Data are collected in a standardized way in all hospitals involved in the register. Procedures and definitions of the items were set down in a manual. 13 hospitals of the BSR participated in a separate follow-up study of patients 3 months after stroke. Details on methods and conduct of the follow-up have been published previously [23]. Briefly, patient recruitment took part between June 2010 and September 2011. Study participants received a postal questionnaire 3 months after stroke or were offered to answer the questions on the phone. If participants were unable to answer the questionnaire or unable to be interviewed due to cognitive impairment or communication difficulties, the questions could also be answered by proxies. Data collected in the follow-up included information about recurrent events, living situation, utilization of rehabilitation services, current medication as well as socioeconomic variables like formal education and immigration background and functional impairment. Impairment was assessed using the Barthel index. Reliability of the postal version of the BI and the telephone version with either patients or carers 3 months after stroke has been demonstrated to be good to excellent when compared to face-to-face interviews [24].

### Study population

Patients who were admitted to one of the participating hospitals after first or recurrent acute stroke or TIA within 7 days after onset of symptoms and aged ≥18 years were included in the register. Patients who were alive at discharge from hospital who gave informed consent to participate in the study were followed up 3 months after stroke. The following analyses were restricted to patients with ischemic stroke as main diagnosis at discharge. Patients with TIA, intracerebral or subarachnoid haemorrhage were excluded.

### Definition of variables

#### Baseline characteristics

During stay in hospital data were collected on the following clinical characteristics: Severity of neurological deficit according to the German version of the National Institutes of Health Stroke Scale (NIHSS)[25], functional impairment at stroke onset using the Barthel Index [24], comorbidities (diabetes mellitus [elevated blood glucose level or history of diabetes or medical treatment], atrial fibrillation [documented by standard-ECG or long-term-ECG or history of atrial fibrillation or medical treatment], previous stroke [evidence for previous stroke lasting >24 hours in patient record or imaging], hypertension [systolic blood pressure of >140 mm Hg or diastolic blood pressure of >90 mm Hg or medical treatment] and hypercholesterolemia [elevated cholesterol level (>190 mg/dL) or history of hypercholesterolemia or medical treatment]. Age and sex of the patients as well as pre-stroke dependency, defined as independent at home, nursing care at home or living in a nursing home) were also documented by members of hospital staff.

#### Early complications

We acquired information on the occurrence of early in-hospital complications, namely on pneumonia [clinical or diagnostic findings of hospital-acquired pulmonary infection], increased intracranial pressure [imaging evidence of cerebral oedema or brain shift syndrome with clinical deterioration]. Complications like urinary tract infection, intracerebral bleeding, recurrent stroke, epileptic seizures, thrombosis or pulmonary embolism and others were rated as complications if they required diagnostic or therapeutic measures. Within the current analysis, the cumulative number of in-hospital complications was used to examine their influence on patient outcome.

#### Mortality and poor outcome 3 months after stroke

Poor outcome 3 months after stroke was defined as death, institutionalization due to stroke or dependency (defined as Barthel Index < 60 points) based on previously published recommendations.[26] Mortality was assessed using information from the registration office which keeps records of the vital status and date of death of all registered inhabitants. Functional impairment was defined by the Barthel Index.[25] The instrument assesses limitations in activities of daily living (ADL) on a score from 0 (completely dependent) to 100 (independent). Institutionalization was defined as the new requirement for nursing home care if patients had previously been living in their own home.

### Statistical analysis

Descriptive statistics were used to provide information on the patients’ baseline characteristics and to assess mortality and poor outcome 3 months after stroke.

Univariate logistic regression analyses were performed to estimate the effect of early complications and of clinical or demographic factors on 3-months mortality and poor outcome. To estimate odds ratios (OR) and resulting 95% confidence intervals (CI) of poor outcome or mortality three months after stroke, we performed multivariable logistic regression analyses including the number of early complications, age, sex, pre-stroke dependency, comorbidities and stroke severity as independent variables. Variables were eliminated using backward selection methods. We calculated attributable fractions (AFs) to examine the relevance of the factors included in the model in terms of mortality and poor outcome after stroke. Attributable fractions are defined as the proportional reduction of an outcome if the exposure to a risk factor was reduced to zero. They consider not only the association between exposure and disease, but also the frequency of the exposure in the population and thus help to estimate the risk factor’s relevance on the population level. The method of average sequential attributable fractions applied allows obtaining estimates for several risk factors which do not add up to more than 100% directly from logistic regression analyses and the attributable fractions are independent from the order in which they are inserted.[4,27] AFs were calculated for all variables included in the final logistic regression model. The independent variables were dichotomized before being included in the model.

To examine whether the estimates found in the study might be modified by selection bias we compared the baseline characteristics of patients who participated in the follow-up with those of the total population documented in the Berlin Stroke Register (BSR). We used the chi-squared test for categorical data to analyse whether age, stroke severity and functional impairment at stroke onset were evenly distributed across these groups. All tests were two-sided, and statistical significance was determined at an alpha level of 0.05. Statistical analyses were performed with the SPSS Statistics 18.0 and SAS 9.2 software package.

### Ethics approval

Patients or their legal representatives provided written informed consent to participate in the study. The study has been approved by the ethics committee of Charité – Universitätsmedizin Berlin (EA1/061/10). The data management was approved by the corresponding data protection officer of the participating institutions.

## Results

**Figure 1:**
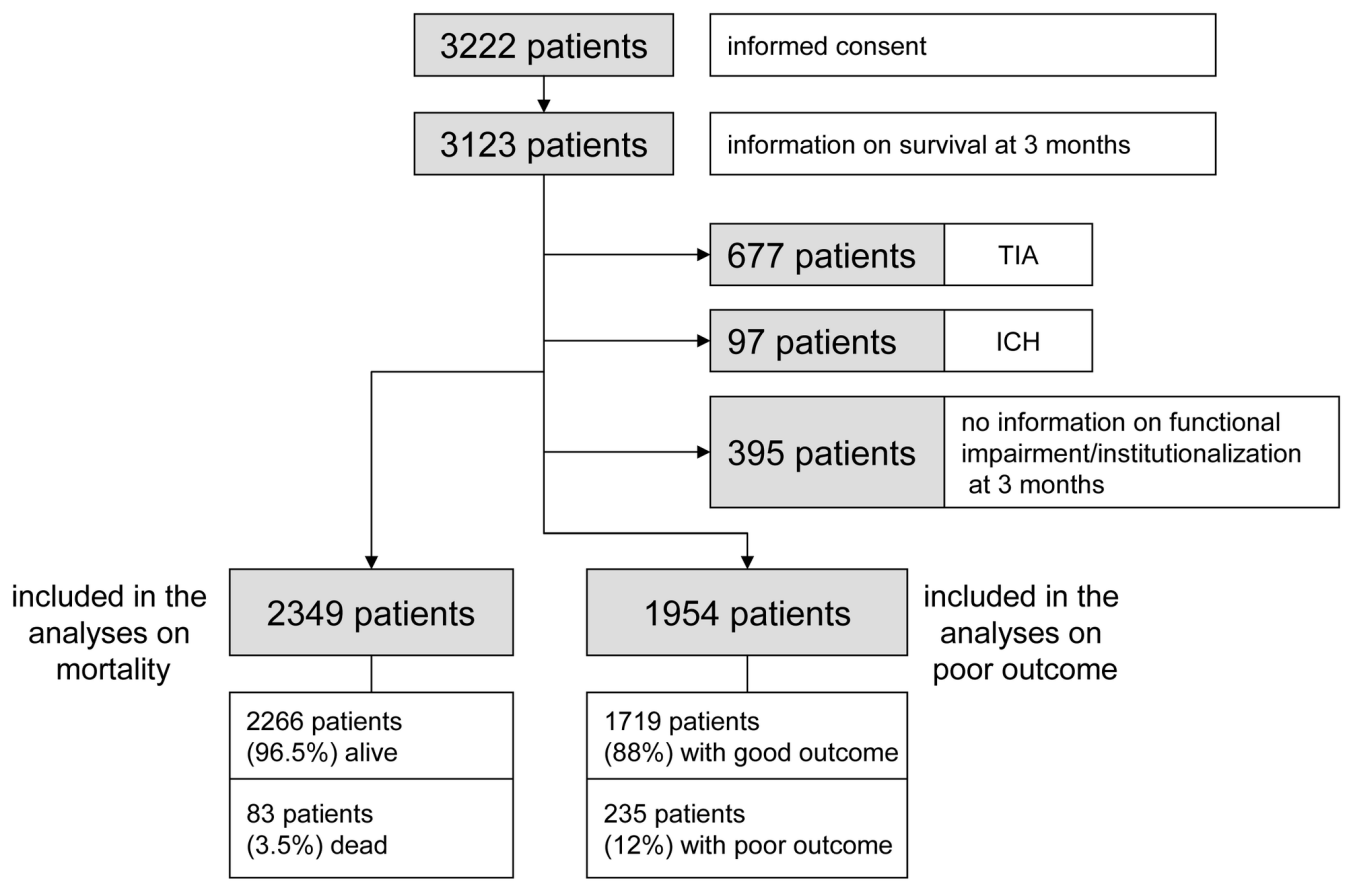
Flow chart of the study population.

Between June 2010 and September 2011, 3222 patients alive at discharge gave informed consent to participate in the follow-up study, giving a participation rate of 64.9% of all patients screened in the participating hospitals during the study period [Figure 1]. Of all patients alive at discharge who agreed to participate in the follow-up, information on survival 3 months after stroke was available for a total of 3123 patients (96.9%). We excluded patients with a final diagnosis of TIA (677 patients) or ICH (97 patients) leaving 2349 patients who were included in the analyses on 3-months case fatality. Mortality of patients with ischemic stroke between discharge from hospital and 3 months after stroke was 3.5% (83 patients). 395 patients (16.8%) could not be included in the analyses on poor outcome 3 months after stroke because of missing information on functional impairment or current living situation. Therefore, 1954 patients with complete follow-up were included in further analyses. Of those, 235 patients (12%) had a poor outcome in terms of death, institutionalization or BI<60.

Baseline characteristics of the 2349 patients with information on survival are presented in Table 1. Of the patients, 60% were <75 years of age and 43.2% were females. Median NIHSS score on admission of the patients was 3 (interquartile range 2 to 6). Functional impairment at stroke onset was severe or moderate (BI 0-95) in 59% of the patients. Early medical complications during stay in hospital occurred in 285 patients (12.1%), in 47 patients (2.0%) at least two complications were documented. The most common complications were urinary tract infection (3.6%), pneumonia (2.3%) and recurrent strokes (1.0%).

**Table 1:** Baseline characteristics of the study population (n = 2349).

Age group, y, n (%)	
<65	704 (30.0)
65-74	702 (29.9)
75-84	664 (28.3)
≥85	279 (11.9)
Female sex, n (%)	1015 (43.2)
Pre-stroke disability	
Home independent	2034 (86.6)
Nursing care at home	197 (8.4)
Nursing care in institution	94 (4.0)
NIHSS categories, n (%)	
<5	1485 (63.2)
5-15	750 (31.9)
>16	111 (4.7)
BI at stroke onset, n (%)	
Poor/moderate (0-95)	1363 (58.8)
Good (100)	955 (40.7)
Comorbidities	
Diabetes	674 (28.7)
Atrial fibrillation	552 (23.5)
Previous stroke	541 (23.0)
Hypertension	1937 (82.5)
Hypercholesterolemia	1401 (59.6)
Complications	
Pneumonia	53 (2.3)
Re-infarct	23 (1.0)
Urinary tract infection	84 (3.6)
Increased intracranial pressure	5 (0.2)
Intracerebral bleeding	16 (0.7)
Epileptic seizures	20 (0.9)
Thrombosis / pulmonary embolism	10 (0.4)
Other complications	126 (5.4)
Number of complications	
0	2064 (87.9)
1	238 (10.1)
≥2	47 (2.0)

We examined differences in relevant determinants of mortality and poor outcome between our study sample and the total population of ischemic stroke patients documented in the Berlin Stroke Register (BSR) alive at discharge between 06/2010 and 09/2011. Patients who participated in the follow-up were significantly younger, less frequently dependent before stroke, had less severe strokes and minor functional impairment at stroke onset and less complications documented during stay in hospital [data not shown].

### Univariate analyses

Unadjusted analyses showed that the cumulative number of complications was associated with higher case-fatality, which also holds true for pneumonia and urinary tract infection, but not for recurrent strokes, when included as distinct variables [see Table 2]. Patients alive 3 months after stroke were also significantly younger and much more likely to have had a milder stroke than patients who had died. Case-fatality was significantly higher among patients dependent on nursing care (at home or in an institution) before stroke. Diabetes and atrial fibrillation were also associated with higher mortality; sex, however, did not have a significant effect on case-fatality.

**Table 2:** Univariate analyses: Mortality and poor outcome 3 months after stroke.

	Mortality n=2349			Poor outcome n=1954		
	% Dead	OR	95% CI	% poor outcome	OR	95% CI
Total	3.5			12.0		
Age group, y						
<65	0.9	1.00		4.4	1.00	
65-74	1.9	2.20	0.83-5.81	7.8	1.84	1.11-3.03
75-84	5.3	6.47	2.71-15.49	16.7	4.38	2.77-6.92
≥85	10.4	13.50	5.54-32.89	31.8	10.16	6.21-16.63
Sex						
Male	2.9	1.00		9.7	1.00	
Female	4.3	1.51	0.97-2.33	15.2	1.67	1.27-2.20
Pre-stroke disability						
Home independent	2.4	1.00		8.1	1.00	
Nursing care at home	9.6	4.32	2.49-7.51	37.3	6.74	4.60-9.87
Nursing care in institution	16.0	7.69	4.14-14.31	60.3	17.18	10.26-28.77
NIHSS categories						
<5	1.7	1.00		5.5	1.00	
5-15	5.7	3.55	2.15-5.86	22.8	5.09	3.74-6.93
16-25	14.2	9.63	4.91-18.89	38.0	10.53	6.30-17.60
>25	0	-	-	50.0	17.20	2.39-123.9
Comorbidities (n/y)						
Diabetes						
no	2.8	1.00		10.1	1.00	
yes	5.3	1.94	1.25-3.03	17.1	1.84	1.39-2.44
Atrial fibrillation						
no	2.6	1.00		9.3	1.00	
yes	6.5	2.59	1.66-4.04	20.9	2.56	1.93-3.40
Previous stroke						
no	3.2	1.00		9.6	1.00	
yes	4.8	1.54	0.96-2.48	20.7	2.48	1.86-3.30
Hypertension						
no	2.0	1.00		7.6	1.00	
yes	3.9	2.02	0.97-4.22	13.0	1.81	1.18-2.76
Hypercholes-terolemia						
no	3.5	1.00		12.6	1.00	
yes	3.5	0.99	0.63-1.56	11.7	0.92	0.70-1.22
No. of complications						
0	2.4	1.00		9.2	1.00	
1	10.1	4.52	2.72-7.50	31.0	4.42	3.10-6.29
≥2	19.1	9.54	4.38-20.79	52.9	10.55	5.54-22.26
Pneumonia		5.27	2.40-11.56		4.76	2.51-9.01
Re-infarcts		2.64	0.61-11.45		6.70	2.56-17.53
Urinary tract infections		5.74	3.04-10.86		10.30	5.96-17.76

The number of complications had a strong influence on poor outcome at 3 months in univariate analyses. Included as distinct variables, pneumonia, urinary tract infection and recurrent strokes all showed a statistically significant association with poor outcome. Higher odds for poor outcome were also observed for older age, female sex, stroke severity and pre-stroke dependency and for diabetes, atrial fibrillation, hypertension and previous strokes.

### Multivariable analyses

In multivariable analyses [Table 3], the number of complications, older age, stroke severity and pre-stroke dependency remained independent predictors of mortality 3 months after stroke.

**Table 3:** Multivariable analyses: Mortality and poor outcome 3 months after stroke.

	Mortality			Poor outcome		
	OR	95% CI	P value	OR	95% CI	P value
Age group, y			<0.005			<0.005
<65	1.00			1.00		
65-74	1.89	0.71-5.06		1.47	0.86-2.51	
75-84	4.37	1.79-10.65		2.86	1.74-4.69	
≥85	6.76	2.66-17.20		4.40	2.52-7.69	
Pre-stroke disability			0.016			<0.005
Home independent	1.00			1.00		
Nursing care at home	2.27	1.25-4.13		3.63	2.36-5.60	
Nursing care in institution	2.43	1.19-4.95		6.79	3.72-12.39	
NIHSS categories			<0.005			<0.005
<5	1.00			1.00		
5-15	2.22	1.31-3.77		3.41	2.43-4.79	
16-25	4.83	2.27-10.28		6.55	3.54-12.12	
>25	0.0	-		4.38	0.28-68.70	
Diabetes	1.60	0.99-2.59	0.056	1.39	0.99-1.94	0.057
Previous strokes				1.99	1.41-2.80	<0.001
No. of complications			<0.001			<0.005
0	1.00			1.00		
1	2.48	1.44-4.28		2.50	1.65-3.79	
≥2	4.45	1.86-10.64		4.92	2.19-11.05	

In-hospital complications, older age, stroke severity, pre-stroke dependency and previous strokes were found to be predictors of poor outcome at 3 months.

Patients with documented complications during acute treatment had more than two-fold increased odds of death (OR 2.48; 95% CI 1.44-4.28) or poor outcome (OR 2.50; 95% CI 1.65-3.79) 3 months after stroke. Case fatality of patients with two or more complications was even higher (OR 4.45; 95% CI 1.86-10.64) as were the odds of poor outcome (OR 4.92; 95% CI 2.19-11.05).

In a separate sensitivity analysis including infections (pneumonia and urinary tract infections) and recurrent strokes as distinct complications we found a significant effect on 3 months mortality for infections (OR 1.93; 95% CI 1.03-3.63), but not for recurrent strokes. Having experienced an infection (OR 3.03; 95% CI 1.78-5.17) or recurrent stroke (OR 12.21; 95% CI 3.83-38.96) had a substantial impact on poor outcome at 3 months.

### Attributable fractions

Attributable fractions for death and poor outcome 3 months after stroke can be seen in Table 4. More than 60% of deaths after discharge from hospital were attributed to age (31.4%), pre-stroke dependency (11.0%) and stroke severity (22.3%). Attributable risks of these factors on poor outcome at 3 months were similar (overall 63.5%) with age having a smaller (21%) and stroke severity a larger effect (27.5%). The proportion of patients with poor outcome attributable to pre-stroke dependency was slightly higher than the proportion of death attributable to this factor (15% vs. 11%).

**Table 4:** Attributable risks of deaths and poor outcome 3 months after ischemic stroke.

	Mortality	Poor outcome at 3 months
Age ≤ 75 y	31.4	21.0
Pre-stroke dependency	11.0	15.0
NIHSS score ≥ 5	22.3	27.5
Diabetes mellitus	6.5	4.0
Previous strokes	NS	7.7
No. of complications ≥1	12.3	9.1
*Total explained*	*83.5*	*84.3*

In-hospital complications contributed to 12.3% of deaths during follow-up, the impact of complications on poor outcome was slightly smaller (9.1%). An additional proportion of 7.7% of poor outcomes could be attributed to previous strokes.

## Discussion

In this study we analysed the independent influence of early in-hospital complications on mortality and dependency 3 months after stroke in ischemic stroke patients alive at discharge from hospital. We demonstrated that the occurrence of early complications has a substantial impact on patient outcome not only in the acute phase after stroke, but also in the 3 months after the event. We noticed a cumulative effect with patients suffering from more than one complication showing significantly higher rates of both case-fatality and poor outcome than patients with only one complication. At 12.3% and 9.1% respectively, a considerable proportion of death and poor outcome in the period after hospital discharge could be attributed to in-hospital complications. A strong influence of complications on early stroke outcome during hospitalisation has previously been reported. A prior analysis from the Berlin Stroke Register demonstrated that odds for in-hospital death were more than 5 times increased for patients with pneumonia, more than 30 times increased for patients with increased intracranial pressure and more than 6 times increased for patients with other in-hospital complications [4]. Consistent with previous studies we found that older age, severity of neurological deficit at stroke onset and pre-stroke dependency are independent predictors of mortality and poor outcome [1,13,28]. In contrast to the results of previous studies [1,13,17] the presence of comorbidities other than previous strokes did not have an independent influence on outcome in our study sample.

Prior studies on the influence of early complications on patient outcome mainly focus on the acute stroke phase, however, little is known about their impact on longer-term stroke outcome. Only few studies analyzed the influence of early complications on outcome 3 months after ischemic stroke [19-21,29].

They demonstrated a similar increase in risk of poor outcome and death in patients who experienced early complications like urinary tract infections and pneumonia as we discovered in our data, with patients experiencing multiple complications carrying a higher risk of death than patients with only one complication [20]. Another study reported an increased 1-year mortality rate for patients experiencing early complications after stroke, particularly pneumonia [14].

In our study, we found that early complications account for an additional 12.3% of deaths occurring between discharge and 3 months after stroke and for an additional 9.1% of poor outcomes at 3 months. This result is in line with prior analyses of the BSR, which demonstrated that early complications (in particular pneumonia, iICP, and others) accounted for 33% and 29% of in-hospital mortality in patients with lengths of stay (LOS) in hospital ≤7 days and >7 days, respectively. Complications also accounted for 15% of poor outcomes (patients with LOS ≤7 days) and 13% of poor outcomes (patients with LOS >7 days) at discharge from hospital [4]. Our results thus confirm the assumption that, although their impact on early outcome seems to be greatest, having experienced in-hospital complications does not only affect short-term patient outcome after stroke, but may also have serious consequences several months after discharge from hospital.

We aimed to estimate the impact of early complications on patient outcome since medical complications after stroke, as opposed to other determinants of outcome, are potentially modifiable, although not completely avoidable. Our results, together with previous literature, underline the need to improve prevention, early detection and treatment of medical complications in stroke units. A prophylactic treatment to prevent complications before they might occur has been suggested as a potential third pillar to improve stroke outcome besides acute treatment and secondary prevention [16]. The implementation of evidence-based management protocols for the treatment of complications in acute stroke units has recently been shown to positively affect patient outcome. Early mobilization of patients after ischemic stroke might also prove helpful to reduce the risk of medical complications [30]. However, despite the fact that it might be possible to significantly reduce the occurrence of early post-stroke complications in hospital, deviations from evidence-based care might also reflect patient and family preferences to withhold or withdraw interventions that increase chances of survival. A recent study estimated that up to 40% of inpatient stroke mortality might be attributed to deliberate decisions against life-sustaining measures [31].

Our study has strengths and limitations. The strengths of the study are its large sample size and its multicentre approach. The study’s limitations include a participation rate of 64.9%. In 20% of the participants we obtained information on survival only. Information on function before hospitalization could be estimated only roughly using the patients’ living situation before stroke as a proxy. Having more detailed information on patients’ pre-stroke status would be highly desirable. The occurrence of in-hospital complications in our sample is lower than complication rates reported in the previous study [4]. This is mainly due to the fact that we included only patients who were alive at discharge from hospital. Since early complications are a major factor influencing in-hospital death our estimates for incident complications are considerably decreased by not including patients who died during stay in hospital. This restriction also means our data allow conclusions only on the effect of early complications on patients who survived the acute phase after stroke, not on their overall impact on 3 months outcome. A comparison of our study population to all registered patients alive at discharge from hospital showed that patients in our sample were significantly younger, had a better pre-stroke condition and were less severely affected, thus suggesting the presence of selection bias in our sample. This might further help to explain the reduced incidence of complications in our study population. However, even in a sample of less affected stroke patients we could demonstrate a strong impact of in-hospital complications on both mortality and poor outcome 3 months after stroke.

## Conclusion

With the majority of deaths and poor outcomes in ischemic stroke patients alive at hospital discharge being attributable to non-modifiable risk factors like age and stroke severity, early complications have been shown to significantly affect patient outcome not only in the short term, but also several months after acute treatment. Some of these complications might be preventable or can be effectively treated if detected early. Early diagnosis of complications as well as evidence-based management is of major importance to reduce their potential consequences. A stronger focus of further research activities on these potentially modifiable determinants of stroke outcome and on efforts to improve interventions to prevent and treat post-stroke complications is therefore required.

## References

[1] Andersen KK, Andersen ZJ, Olsen TS (2011) Predictors of early and late case-fatality in a nationwide Danish study of 26,818 patients with first-ever ischemic stroke. Stroke 42: 2806-2812. doi:10.1161/STROKEAHA.111.619049. PubMed: 21817152. 21817152 10.1161/STROKEAHA.111.619049

[2] Bravata DM, Ho SY, Brass LM, Concato J, Scinto J et al. (2003) Long-term mortality in cerebrovascular disease. Stroke 34: 699-704. doi:10.1161/01.STR.0000057578.26828.78. PubMed: 12624294. 12624294 10.1161/01.STR.0000057578.26828.78

[3] Ingeman A, Pedersen L, Hundborg HH, Petersen P, Zielke S et al. (2008) Quality of care and mortality among patients with stroke: a nationwide follow-up study. Med Care 46: 63-69. doi:10.1097/MLR.0b013e3181484b91. PubMed: 18162857. 18162857 10.1097/MLR.0b013e3181484b91

[4] Koennecke HC, Belz W, Berfelde D, Endres M, Fitzek S et al. (2011) Factors influencing in-hospital mortality and morbidity in patients treated on a stroke unit. Neurology 77: 965-972. doi:10.1212/WNL.0b013e31822dc795. PubMed: 21865573. 21865573 10.1212/WNL.0b013e31822dc795

[5] Palnum KD, Andersen G, Ingeman A, Krog BR, Bartels P et al. (2009) Sex-related differences in quality of care and short-term mortality among patients with acute stroke in Denmark: a nationwide follow-up study. Stroke 40: 1134-1139. doi:10.1161/STROKEAHA.108.543819. PubMed: 19211479. 19211479 10.1161/STROKEAHA.108.543819

[6] Schneider K, Heise M, Heuschmann P, Berger K (2009) Lebens- und Versorgungssituation von Schlaganfallpatienten. 3-Monats-Follow-up des Qualitätssicherungsprojektes Nordwestdeutschland. Nervenheilkunde 28: 114-118.

[7] Kolominsky-Rabas PL, Heuschmann PU (2002) [Incidence, etiology and long-term prognosis of stroke]. Fortschr Neurol Psychiatr 70: 657-662. doi:10.1055/s-2002-35857. PubMed: 12459947. 12459947 10.1055/s-2002-35857

[8] Ward A, Payne KA, Caro JJ, Heuschmann PU, Kolominsky-Rabas PL (2005) Care needs and economic consequences after acute ischemic stroke: the Erlangen Stroke Project. Eur J Neurol 12: 264-267. doi:10.1111/j.1468-1331.2004.00949.x. PubMed: 15804242. 15804242 10.1111/j.1468-1331.2004.00949.x

[9] Heuschmann PU, Wiedmann S, Wellwood I, Rudd A, Di Carlo A et al. (2011) Three-month stroke outcome: the European Registers of Stroke (EROS) investigators. Neurology 76: 159-165. doi:10.1212/WNL.0b013e318206ca1e. PubMed: 21148118. 21148118 10.1212/WNL.0b013e318206ca1e

[10] Grau AJ, Weimar C, Buggle F, Heinrich A, Goertler M et al. (2001) Risk factors, outcome, and treatment in subtypes of ischemic stroke: the German stroke data bank. Stroke 32: 2559-2566. doi:10.1161/hs1101.098524. PubMed: 11692017. 11692017 10.1161/hs1101.098524

[11] Hankey GJ (2003) Long-term outcome after ischaemic stroke/transient ischaemic attack. Cerebrovasc Dis 16 Suppl 1: 14-19. doi:10.1159/000069936. PubMed: 12698014. 10.1159/000069936 12698014

[12] Appelros P, Nydevik I, Viitanen M (2003) Poor outcome after first-ever stroke: predictors for death, dependency, and recurrent stroke within the first year. Stroke 34: 122-126. doi:10.1161/01.STR.0000047852.05842.3C. PubMed: 12511762. 12511762 10.1161/01.str.0000047852.05842.3c

[13] Saposnik G, Hill MD, O’Donnell M, Fang J, Hachinski V et al. (2008) Variables associated with 7-day, 30-day, and 1-year fatality after ischemic stroke. Stroke 39: 2318-2324. doi:10.1161/STROKEAHA.107.510362. PubMed: 18566303. 18566303 10.1161/STROKEAHA.107.510362

[14] Ingeman A, Andersen G, Hundborg HH, Svendsen ML, Johnsen SP (2011) In-hospital medical complications, length of stay, and mortality among stroke unit patients. Stroke 42: 3214-3218. doi:10.1161/STROKEAHA.110.610881. PubMed: 21868737. 21868737 10.1161/STROKEAHA.110.610881

[15] Middleton S, McElduff P, Ward J, Grimshaw JM, Dale S et al. (2011) Implementation of evidence-based treatment protocols to manage fever, hyperglycaemia, and swallowing dysfunction in acute stroke (QASC): a cluster randomised controlled trial. Lancet 378: 1699-1706. doi:10.1016/S0140-6736(11)61485-2. PubMed: 21996470. 21996470 10.1016/S0140-6736(11)61485-2

[16] Minnerup J, Schäbitz WR (2012) Improving outcome after stroke: time to treat new targets. Stroke 43: 295-296. doi:10.1161/STROKEAHA.111.642363. PubMed: 22198977. 22198977 10.1161/STROKEAHA.111.642363

[17] Kumar S, Selim MH, Caplan LR (2010) Medical complications after stroke. Lancet Neurol 9: 105-118. doi:10.1016/S1474-4422(09)70266-2. PubMed: 20083041. 20083041 10.1016/S1474-4422(09)70266-2

[18] Balami JS, Chen RL, Grunwald IQ, Buchan AM (2011) Neurological complications of acute ischaemic stroke. Lancet Neurol 10: 357-371. doi:10.1016/S1474-4422(10)70313-6. PubMed: 21247806. 21247806 10.1016/S1474-4422(10)70313-6

[19] Hong KS, Kang DW, Koo JS, Yu KH, Han MK et al. (2008) Impact of neurological and medical complications on 3-month outcomes in acute ischaemic stroke. Eur J Neurol 15: 1324-1331. doi:10.1111/j.1468-1331.2008.02310.x. PubMed: 19049549. 19049549 10.1111/j.1468-1331.2008.02310.x

[20] Wang PL, Zhao XQ, Yang ZH, Wang AX, Wang CX et al. (2012) Effect of in-hospital medical complications on case fatality post-acute ischemic stroke: data from the China National Stroke Registry. Chin Med J (Engl) 125: 2449-2454. PubMed: 22882920. 22882920

[21] Wang PL, Zhao XQ, Du WL, Wang AX, Ji RJ et al. (2013) In-hospital medical complications associated with patient dependency after acute ischemic stroke: data from the China National Stroke Registry. Chin Med J (Engl) 126: 1236-1241. PubMed: 23557550. 23557550

[22] Koennecke H-C, Walter G (2008) Qualitätssicherung in der Schlaganfallversorgung. Das Berliner Schlaganfallregist Berliner Ärzte 9: 24-25.

[23] Grube MM, Koennecke HC, Walter G, Thümmler J, Meisel A et al. (2012) Association between socioeconomic status and functional impairment 3 months after ischemic stroke: the Berlin Stroke Register. Stroke 43: 3325-3330. doi:10.1161/STROKEAHA.112.669580. PubMed: 23033351. 23033351 10.1161/STROKEAHA.112.669580

[24] Heuschmann PU, Kolominsky-Rabas PL, Nolte CH, Hünermund G, Ruf HU et al. (2005) The reliability of the german version of the barthel-index and the development of a postal and telephone version for the application on stroke patients. Fortschr Neurol Psychiatr 73: 74-82. doi:10.1055/s-2005-870995. PubMed: 15685491. 15685491 10.1055/s-2004-830172

[25] Berger K, Weltermann B, Kolominsky-Rabas P, Meves S, Heuschmann P et al. (1999) The reliability of stroke scales. The german version of NIHSS, ESS and Rankin scales. Fortschr Neurol Psychiatr 67: 81-93. doi:10.1055/s-2007-993985. PubMed: 10093781. 10093781 10.1055/s-2007-993985

[26] Sulter G, Steen C, De Keyser J (1999) Use of the Barthel index and modified Rankin scale in acute stroke trials. Stroke 30: 1538-1541. doi:10.1161/01.STR.30.8.1538. PubMed: 10436097. 10436097 10.1161/01.str.30.8.1538

[27] Rückinger S, von Kries R, Toschke AM (2009) An illustration of and programs estimating attributable fractions in large scale surveys considering multiple risk factors. BMC Med Res Methodol 9: 7. doi:10.1186/1471-2288-9-7. PubMed: 19166593. 19166593 10.1186/1471-2288-9-7 PMC2636839

[28] Koton S, Tanne D, Green MS, Bornstein NM (2010) Mortality and predictors of death 1 month and 3 years after first-ever ischemic stroke: data from the first national acute stroke Israeli survey (NASIS 2004). Neuroepidemiology 34: 90-96. doi:10.1159/000264826. PubMed: 20016218. 20016218 10.1159/000264826

[29] Johnston KC, Li JY, Lyden PD, Hanson SK, Feasby TE et al. (1998) Medical and neurological complications of ischemic stroke: experience from the RANTTAS trial. RANTTAS Investigators. Stroke 29: 447-453. doi:10.1161/01.STR.29.2.447. PubMed: 9472888. 9472888 10.1161/01.str.29.2.447

[30] Diserens K, Moreira T, Hirt L, Faouzi M, Grujic J et al. (2012) Early mobilization out of bed after ischaemic stroke reduces severe complications but not cerebral blood flow: a randomized controlled pilot trial. Clin Rehabil 26: 451-459. doi:10.1177/0269215511425541. PubMed: 22144725. 22144725 10.1177/0269215511425541

[31] Kelly AG, Hoskins KD, Holloway RG (2012) Early stroke mortality, patient preferences, and the withdrawal of care bias. Neurology 79: 941-944. doi:10.1212/WNL.0b013e318266fc40. PubMed: 22927679. 22927679 10.1212/WNL.0b013e318266fc40 PMC3425847

